# Development and validation a task-specific checklist for a microsurgical varicocelectomy simulation model

**DOI:** 10.1590/S1677-5538.IBJU.2019.0571

**Published:** 2020-07-31

**Authors:** Marcelo Esteves Chaves Campos, Marcelo Magaldi Ribeiro de Oliveira, Augusto Barbosa Reis, Lilian Bambirra de Assis, Viacheslav Iremashvili

**Affiliations:** 1 Universidade Federal de Minas Gerais - UFMG Departamento de Cirurgia Belo Horizonte MG Brasil Departamento de Cirurgia, Universidade Federal de Minas Gerais - UFMG, Belo Horizonte, MG, Brasil; 2 CEFET Departamento de Ciências Sociais Aplicadas Belo Horizonte MG Brasil Departamento de Ciências Sociais Aplicadas, CEFET, Belo Horizonte, MG, Brasil; 3 United Hospital Center Department of Urology Bridgeport West Virginia USA, United States Department of Urology, United Hospital Center, Bridgeport, West Virginia, USA, United States

**Keywords:** Simulation Training, Educational Measurement, Microsurgery

## Abstract

**Purpose:**

To develop and validate a new test of specific technical skills required for microsurgical varicocelectomy.

**Materials and Methods:**

An electronic questionnaire was sent to 558 members of the Brazilian Society of Urology for the validation of the task-specific checklist (TSC) for assessment of microsurgical varicocelectomy. Participants who had experience in this procedure were selected as judges. For construct validation, 12 participants including attending urologists and urological residents in training were recruited for voluntary participation. We formed a group of three experts and a group of nine novices, who had to perform the steps of microsurgical varicocelectomy on a simulation model using human placenta. Each participant was filmed and two blinded raters would then evaluate their performance using the TSC of microsurgical varicocelectomy.

**Results:**

14 judges were recruited. The assessment tool was reformulated, according to the judges suggestions and had the content validity achieved. The final version of the TSC was comprised of the task-specific score, a series of 4 items scored in a binary fashion designed for microscopic sub-inguinal varicocelectomy. The differences between the performance of participants with different levels of experience reflected the construct validity. The reliability between the raters was high. The mean time required to complete the training of microsurgical varicocelectomy in simulation model was significantly shorter for experts compared to novices (201 vs. 496 seconds, p=0.01).

**Conclusions:**

This preliminary study suggests that the task-specific checklist of microsurgical varicocelectomy is reliable and valid in assessing microsurgical skills.

## INTRODUCTION

Since the classic work of Tulloch, varicoceles are known to be associated with male factor infertility (1). Surgical correction of varicoceles improves the rate of spontaneous pregnancy making this disease the most important surgically correctable cause of infertility in males (2). Some other less common indications for varicocelectomy include testicular pain and testicular dysfunction (3).

Marmar, Debenedictis & Praiss (1985) described the microdissection of the spermatic cord at the external inguinal ring for the management of varicoceles (4). Since then, the use of microsurgical techniques has been widely adopted, improving results and reducing surgical complications by allowing better identification and preservation of lymphatics and testicular arteries (5). Although sub-inguinal microsurgical varicocelectomy is currently considered the gold standard treatment for varicocele, microsurgical manipulations are not parts of the skill set of many urologists making this procedure challenging (6).

The steep learning curve in the acquisition of microsurgical skills defines the need for training outside of the operating room (7). Training in the laboratory on simulation models may help providers develop familiarity with micro instruments handling, as well as cognitive and technical competency in microsurgery (8, 9).

Several models have been proposed in teaching and learning of microsurgery practice (10, 11). While assessment of learning skills and abilities gained by the trainees is imperative (12-14), to our knowledge there are no published studies reporting simulation in evaluation of surgical skills in microsurgical varicocelectomy. The purpose of this study was to fill this gap by developing and validating such a test of specific technical skill for microsurgical varicocelectomy simulation model.

## MATERIALS AND METHODS

This study received an approval from a certified Ethical Board and 12 human placentas were collected. The expectant mothers underwent prenatal infectious evaluation and signed consent for donation of placenta for practice in surgical techniques.

The study was divided into three stages. Firstly, a simulation model for the training of microsurgical varicocelectomy was built. Then, a task-specific checklist for assessment of microsurgical varicocelectomy (TSC) was developed. Lastly, a validation study was carried out to determine the reproducibility, reliability and validity of this tool.

### Simulation model

The average human placenta has a diameter of 17.0 to 19.0cm and a thickness of 2.0 to 3.0cm. The allantoid membrane covers the fetal surface. The umbilical cord usually contains two arteries and one vein and the vessels radiate on the fetal surface with diameters ranging from 1.22 to 12.27mm (15).

Placentas were washed with 0.9% saline to remove any blood from their surfaces. The umbilical cords were shortened to 8cm to allow easy catheterization of the umbilical arteries and vein. A 6 French gauge urinary catheter was used to catheterize the umbilical vessels and a 0.9% saline was used to irrigate the specimen until the vessels were free of blood clots.

**Figure 1:**
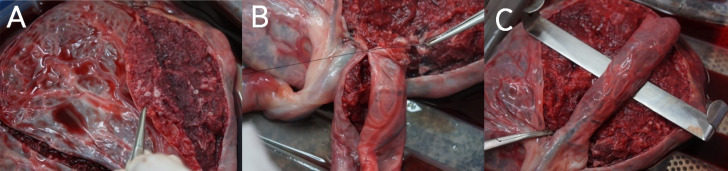
Surgical preparation of human placenta in a varicocele model. A) Cutting 1.0 cm deep and folding the placenta stroma. B) Suturing the folded borders. C) Spermatic funiculus simulation.

Placenta was spread over the operative table, with the fetal surface facing upward. The initial step was to choose a placental vein irrigation area that also included a placental artery to build the simulation model. Cutting 1.0cm deep into the placental stroma around this predetermined region, the placenta was folded inwards to simulate the spermatic funiculus (Figure-1A). This reconstructed funiculus was sutured using a 3-0 Vycril (Figure-1B). The main artery and vein were cannulated with a 6 French gauge urinary catheter and continuous infusion of colored saline solutions (red for artery and blue for vein -0 Gouache 1:10 saline) was started to simulate blood (Figure-1C). Since the placenta vascular tree has just one flow direction, the infused fluid flowed out through the placenta stroma into a bowl connected to a drainage system.

### Development of task-specific checklist

**Figure 2:**
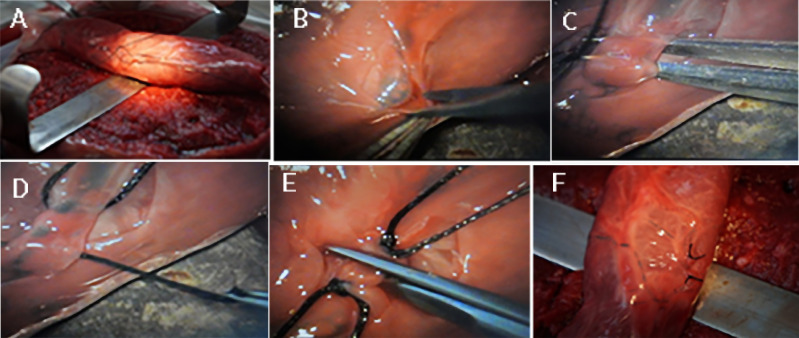
Microsurgical handling of placenta vessels simulating the varicocele treatment. A) Simulated spermatic funiculus put under microscopic working area. B) Allantoic membrane dissection for access to the placenta vessels. C) Placenta vein dissection, after identify artery. D) Placenta vein knot tying. E) Placenta vein micro-scissors cutting. F - Final appearance of vein cutting in the simulated spermatic funiculus.

A task-specific checklist for assessment of microsurgical varicocelectomy was developed, consisting of 7 items scored in a binary fashion (not done/done incorrectly=0 or done correctly=1). These items correspond to important steps of the procedure (Figure-2) and evaluate the technical ability of the provider in performing sub-inguinal microsurgical varicocelectomy. The items were as follows: 1) Keep the sterile field and use the microscope properly; 2) Use the micro-instruments correctly; 3) Recognize and correctly dissect the spermatic fascia for access to the vessels; 4) Correctly identifies arteries and veins; 5) Adequately performs the dissection of the dilated veins; 6) Adequately performs the ligatures of the dilated veins; 7) Adequately performs the dilated veins section.

Then, an electronic questionnaire was sent to 558 members of the Brazilian Society of Urology for the validation of the tool. Participants who performed more than ten microsurgical varicocelectomy per year were selected as judges.

### Validation task-specific checklist

All judges filled out a post-study questionnaire to assess TSC usefulness as an evaluation tool. The items of the test were evaluated (content validity), considering five requirements: pertinence, feasibility, objectivity, clarity and vocabulary. The questionnaires were presented on a 3-point scale (1) Adequate; 2) Adequate with changes; 3) Inadequate). The tool was reformulated, according to the judges suggestions and the final version of the task-specific checklist was created.

For construct validation, 12 participants including urologists and residents in training for urology were recruited for voluntary participation. Two groups were formed based on the microsurgical experience. Group 1 consisted of three urologists who performed more than 100 microsurgical procedures each (Expert group) and group 2 included nine urology residents with little microsurgical experience (performed at most 10 microsurgical procedures; Novice group). The subjects were given a standardized explanation about the surgical steps of the microscopic sub-inguinal varicocelectomy showing a varicocele surgical treatment video and then administered a practice round in the simulation model. Each participant wore a surgical hair net, a face mask and a surgical gown. Each urologic surgery was filmed, with special attention to camera framing so as to film only the hands of the operator during the maneuvers, for anonymization purposes. The videos were then viewed by two educational experts who were unaware of the group assignment. The two education experts also had experience in microsurgical varicocelectomy, but were different from the judges. They rated the participants in-dependently. To evaluate the construct validity, they used the final version of the TSC to compare the performance of participants with different levels of experience. The time required to complete the activity was also measured and compared between the groups.

## RESULTS

Of the 558 questionnaires sent, we received 49 responses eight of which were incomplete. Of the 41 eligible responses, only 14 participants had the surgical microscope available and used it to perform varicocelectomy. These were recruited as judges.

Assessment of content validity of the TSC resulted in five out of 7 items being considered Adequate or Adequate with changes by all 14 judges. The other two items (6 and 7) were evaluated as Inadequate by a single judge.

The judges felt that some items were redundant and should be merged to facilitate the assessments. They also suggested that the handling of microsurgical instruments should not be assessed separately and that this skill should be evaluated throughout all tasks. The tool was reformulated, according to the judges suggestions and reduced to four items. Table-1 presents the final version of the TSC was comprised of the task-specific score, a series of 4 items scored in a binary fashion designed for microscopic sub-inguinal varicocelectomy.

**Table 1:** Final version of the task-specific checklist for microsurgical varicocelectomy.

**INSTRUCTIONS TO PARTICIPANTS** You have just come across a varicocelectomy simulation model built using human placenta. The operating microscope was taken to the surgical field. Under a magnification of 12 x, you should open the allantoid membrane, simulating the spermatic fascia, and identify the artery and the veins. Then you should dissect, perform a double ligation and cut a vein.			
**CHECKLIST**			
**ITEM**		**NOT DONE/ DONE INCORRECTLY**	**DONE CORRECTLY**
1	Adjusts, position and correctly handles the microscope, keeping the sterile field?	0	1
2	Properly recognizes and dissects fascia for access to the vessels?	0	1
3	Adequately performs the dissection of the dilated veins, differentiating them from the arteries and lymph vessels?	0	1
4	Adequately performs vein ligatures and vein section?	0	1
**TOTAL SCORE**		/ 4	
**TIME NEEDED TO COMPLETE**		**SECONDS**	

**Figure 3:**
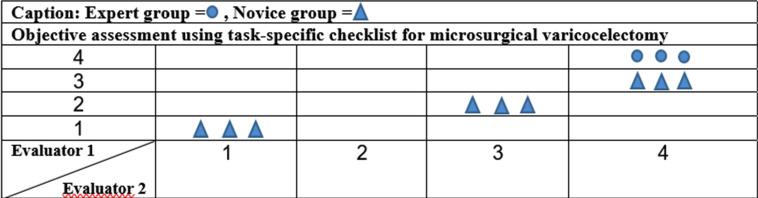
Concordance of the scores between the Educational Experts and construct validity.

Figure 3 shows the differences between experts and novices reflecting the construct validity. The concordance of the scores between the educational experts was not full due to the item “Properly recognizes and dissects fascia for access to the vessels?”, which had only 50% concordance. However, the other three items had an agreement of 100% in the educational experts scores. Moreover, the reliability between the two educational experts, among the Novice group, for which there were disagreements, was full, since both returned the same rank of the nine novices. The mean time required to complete the training of microsurgical varicocelectomy in simulation model was significantly shorter for experts compared to novices (201 vs. 496 seconds, p=0.01).

## DISCUSSION

Microsurgical correction is the standard treatment for varicocele, however familiarity of the urologists with the technical skills necessary for this procedure remains limited (16). The traditional learning model, in which the apprentice observes, assists and finally operates under the supervision of the tutor, requires a long period of training in order to reach the expertise (17). In the modern surgical era simulation training models are becoming a crucial step in the progress of the apprentice towards performing live operations (18). Human placenta was described as a training tool in microsurgery in 1979 when it was used for cutting and suturing vessels without any previous preparation (19). The simulation model used in this study reproduces anatomical conditions encountered during the microsurgical varicocelectomy and recreates a clinical experience without risking patient’s health. As important as the teaching method is the assessment of the learning abilities and skills acquired by the trainees (20). This is the first study that developed and validated a task-specific checklist for assessment of microsurgical varicocelectomy.

The use of an electronic questionnaire in the initial phase of validation of the tool offers many advantages including low cost and user-friendly format easing the recruitment of judges. The main downside is the low response rate. According to Dainesi & Goldbaum (2012), the average response rate of the questionnaires via e-mail is 8.2% (21) which is similar to 8.8% observed in our study.

The high concordance between the judges in the evaluation of the TSC items for the assessments considered Adequate or Adequate/Adequate with changes supports the content validity of the tool. Evaluation of the construct validity demonstrated clear performance differences between the experts and novices. The longer time required by novices to perform the tasks suggests that the model may be used to evaluate and improve microsurgical skills required for the actual procedure, although this remains to be proven.

The concordance measures how often the two educational experts attribute exactly the same score, while the reliability measures the relative similarity between the two sets of ratings. There was disagreement between the two raters among the Novice group in the item “Properly recognizes and dissects fascia for access to the vessels?”, with consequent leveling of the skills of three novices with that of the experts by evaluator 2. Despite this, the reliability between the two educational experts remained high. The vessels in the spermatic funiculus are surrounded by fascia and fatty tissue. In the placenta model they are surrounded by allantoid membrane and placenta stroma. Thus, one of the possible explanations for the discordance for the item “Properly recognizes and dissects fascia for access to the vessels?” was the differences between the educational experts in their perception of the similarity between the allantoid of the human placenta and the spermatic fascia.

The main methodological limitations of this research are the single-center design and small number of providers in both expert and novice groups. It should be noted that our ability to recruit participants for the former group was limited by the shortage of urologists with microsurgical skills in Brazil. In addition, another limitation was that the electronic questionnaire for the selection of judges was sent only to a portion of Brazilian urologists, being a convenience sample. However, despite this selection bias, we believe it was a representative sample.

## CONCLUSIONS

This preliminary study suggests that the task-specific checklist of microsurgical varicocelectomy is reliable and valid in assessing surgical skills when used in the settings of human placental simulation model.
